# Identification of *Daboia siamensis* venome using integrated multi-omics data

**DOI:** 10.1038/s41598-022-17300-1

**Published:** 2022-07-30

**Authors:** Thammakorn Saethang, Poorichaya Somparn, Sunchai Payungporn, Sira Sriswasdi, Khin Than Yee, Kenneth Hodge, Mark A. Knepper, Lawan Chanhome, Orawan Khow, Narongsak Chaiyabutr, Visith Sitprija, Trairak Pisitkun

**Affiliations:** 1 Department of Computer Science, Faculty of Science, Kasetsart University, Bangkok, 10900 Thailand; 2 Center of Excellence in Systems Biology, Faculty of Medicine, Chulalongkorn University, Bangkok, 10330 Thailand; 3 Translational Research in Inflammation and Immunology Research Unit (TRIRU), Department of Microbiology, Faculty of Medicine, Chulalongkorn University, Bangkok, 10330 Thailand; 4 Research Unit of Systems Microbiology, Faculty of Medicine, Chulalongkorn University, Bangkok, 10330 Thailand; 5 Center of Excellence in Computational Molecular Biology, Faculty of Medicine, Chulalongkorn University, Bangkok, 10330 Thailand; 6 Research Affairs, Faculty of Medicine, Chulalongkorn University, Bangkok, 10330 Thailand; 7 Chemical Toxicology Research Division, Department of Medical Research, Ministry of Health, Yangon, 11191 Myanmar; 8 Epithelial Systems Biology Laboratory, NHLBI, National Institutes of Health, Bethesda, MD USA; 9 Queen Saovabha Memorial Institute, The Thai Red Cross Society, Bangkok, 10330 Thailand

**Keywords:** Computational biology and bioinformatics, Protein analysis, Genome, Mass spectrometry

## Abstract

Snakebite, classified by World Health Organization as a neglected tropical disease, causes more than 100,000 deaths and 2 million injuries per year. Currently, available antivenoms do not bind with strong specificity to target toxins, which means that severe complications can still occur despite treatment. Moreover, the cost of antivenom is expensive. Knowledge of venom compositions is fundamental for producing a specific antivenom that has high effectiveness, low side effects, and ease of manufacture. With advances in mass spectrometry techniques, venom proteomes can now be analyzed in great depth at high efficiency. However, these techniques require genomic and transcriptomic data for interpreting mass spectrometry data. This study aims to establish and incorporate genomics, transcriptomics, and proteomics data to study venomics of a venomous snake, *Daboia siamensis*. Multiple proteins that have not been reported as venom components of this snake such as hyaluronidase-1, phospholipase B, and waprin were discovered. Thus, multi-omics data are advantageous for venomics studies. These findings will be valuable not only for antivenom production but also for the development of novel therapeutics.

## Introduction

Snakebite is a crucial public health burden in tropical countries, with over one million bites reported annually according to the World Health Organization (WHO)^1–3^. Snakebite envenomation has been classified as a neglected health condition by WHO since it causes around 90,000–137,880 deaths and 1.8–2.7 million injuries per year and access to antivenoms is still obstructed in many developing countries^4,5^. Currently available commercial antivenoms are the intravenous administration of antivenom prepared from hyperimmune serum of horses, sheep or other large domesticated animals^6^. However, a mixture of antibodies contained in the antivenom do not bind with strong specificity to target toxins, which means that severe complications such as chronic disability and amputations can still occur despite treatment^5–7^. Even in cases of successful treatment, the cost of antivenom is expensive. There was a report showing that an antivenom could cost around 640 USD or even 1000 USD per treatment^4,8^. Furthermore, over the last two decades, several primary antivenom producers (such as Syntex, Behringwerke, and Sanofi Paliisteur) have ceased production of antivenoms. This has led to a current crisis in antivenom availability in various regions of the world, creating an important, life-threatening therapeutic gap^9,10^.

The knowledge of venom compositions is fundamental for producing a specific antivenom that has high effectiveness, low side effects, and ease of manufacture^7,8^. Snake venoms are complex mixtures of small molecules and peptides/proteins which have long evolved to help snakes in hunting prey and in protecting themselves from predators. Snake venom compositions vary among different snake species. Even within the same species, venom compositions vary according to age, geographic location, gender, diet, and seasons^11^. Mass spectrometry techniques have been used to generate and analyze snake venom proteome data^6,7,12,13^. Compared to the aforementioned studies, recent advances in mass spectrometry mean that these proteomes can now be analyzed in great depth at high efficiency^3,14^. However, the modern approach requires genomic and transcriptomic data for identification of proteins, unveiling the contributions of gene content and regulation, and interpreting gene expression. To be clear, fully sequenced genomes are critical for interpreting mass spectrometry data^15^. The identified proteins from snake venom will be highly valuable for the production of antivenoms or new toxin inhibitors.

The increase in availability of snake genome sequencing data will facilitate not only the development of antivenom but also the discovery of new therapeutic candidates from snake venom. As of now, several venom-derived therapeutics have been approved by the Food and Drug Administration (FDA) such as Captopril, Tirofiban, Eptifibatide, Defibrase, Hemocoagulase, and Ximelagatran and multiple candidates are now in clinical trials^16^. Venom-derived drugs are being researched for application in the treatment of hypertension, acute coronary syndromes, perioperative bleeding, congestive heart failure, human immunodeficiency virus (HIV), multiple sclerosis, chronic pain treatment, blood loss during vascular trauma, non-compressible hemorrhage, and cancer^3,6,7,17^. In addition, snake venoms are also reported to possess potential therapeutic properties such as antitumor, antimicrobial, anticoagulating, and analgesic activities^18^. In the future, with the complete snake genome databases, it will be possible to identify novel therapeutic/bioactive molecules by scanning through snake genomes for protein coding regions and comparing these proteins against established pharmacophore databases^17^.

Apart from development of antivenoms and discovery of novel therapeutic agents, genome sequencing data is important for evolutionary research. Since the evolutionary origins of snake venom genes are still not fully understood, genome sequences can be used in evolutionary gene loss analysis, identification of pseudogenes, and phylogeny reconstruction^17^. For example, comparative genomics could identify gene loss of several visual pigment genes in some snakes^19^. This loss might relate to the fossorial behavior (digging and living underground) of ancestral snakes, thus causing reduction or loss of eyesight development^20^. Genome sequences are also important for the annotation of gene isoforms, especially toxin gene isoforms. Multiple isoforms of toxin genes have been found and hypothesized to provide broad spectrum toxicity against various prey species, or to serve as a toxin repertoire to prevent prey from developing resistance. In addition, multiple isoforms could relate to potentiation, since a complex composed of multiple toxins is more potent than a single toxin^21^. With the availability of snake genome sequencing data, evolutionary novelties can be discovered.

To this end, the primary objective of this study is to establish a multi-omics approach to identify venom composition of *Daboia siamensis* by incorporating genomics, transcriptomics, and proteomics data. The generated data will be highly valuable for antivenom production, discovery of novel therapeutics, and evolutionary studies. All novelties found from analyses of our initiated snake omics data will directly benefit individuals who are at risk of envenomation from *Daboia siamensis* which is endemic to Thailand and other parts of Southeast Asia as well as indirectly benefit relevant biomedical communities worldwide.

## Methods

### Snake tissue and venom acquisition and processing

Specimens of Thai *Daboia siamensis* were retrieved from the snake farm of Queen Saovabha Memorial Institute (QSMI), the Thai Red Cross Society, Thailand. The experimental protocol was approved by the Ethic Committee of the Queen Saovabha Memorial Institute Animal Care and Use (Approval no. QSMI-ACUC-03-2016) in accordance with the guidelines of the National Research Council of Thailand. This study is reported in accordance with ARRIVE guidelines (https://arriveguidelines.org). Tissue samples were harvested from an adult female snake weighted 0.495 kg with a snout-to-vent length (SVL) of 96 cm and a total length (TOL) of 108.5 cm. Venom gland and crude venom were harvested from an adult male snake weighted 0.375 kg with SVL of 83 cm and TOL of 97 cm. All specimens were captured from Sakaew province, Thailand. Snakes were housed individually in a secure locked plastic cage, equipped with a hiding box and water bowl. They were fed mice once weekly until being experimented. Prior to venom glands and other organs removal, snakes were fully euthanized by a high dose of isoflurane inhalation and were monitored for at least 20 min to the point of loss of reflex. The euthanized snakes were disposed of according to the QSMI guideline.

### Genomic DNA library preparation

Whole genomic library preparation was carried out using the 10x Genomics linked-read technology. High Molecular Weight (HMW) genomic DNA (gDNA) is required for the preparation process. In this study, HMW gDNA was extracted from fresh *Daboia siamensis* liver tissue. First, 500 mg of tissue was harvested and subsequently snapped frozen in liquid nitrogen. Frozen tissue was stored at − 80 °C before proceeding to gDNA library preparation by Novogene Co., Ltd. (China). Prior to the gDNA extraction process, the frozen tissue was kept in a tube and then thawed by placing a tube on ice. The tissue was cut into pieces (~ 25 mg per piece) and placed in 1.5 ml Kimble Kontes Pellet Pestle tubes. These tubes were maintained on ice. The nuclei isolation-digestion and DNA purification processes were achieved as described in the 10x Genomics Technical Note: Sample Preparation Demonstrated Protocol-Rev B (CG000072). The purified gDNA was stored at − 20 °C and quantified using the Qubit dsDNA BR Kit as described in the 10x Genomics Technical Note: Chromium Genome Reagent Kits User Guide-Rev C (CG00022). The acceptable concentration of the diluted DNA is in the range of 0.8–1.2 ng/µl.

### Snake genome sequencing

Purified gDNA was partitioned across Gel Bead-in-Emulsions (GEMs) using GemCode technology. The processes related to GEM generation and incubation, DNA barcoding, and genome library construction are described in the 10x Genomics Technical Note: Chromium Genome Reagent Kits User Guide-Rev C (CG00022). Illumina-ready sequencing libraries were generated using Chromium Genome Chip and Chromium Controller. The resulting libraries were composed of P5 and P7 adaptors, Read 1, 16 bp 10x Barcode, gDNA insert, Read 2, and sample index ready for sequencing process. Read 1 sequence and the 10x Barcode were included during the GEM incubation whereas P5 and P7 primers, Read 2, and sample index were added during the library construction. Genome sequencing was carried out using the Illumina HiseqX system to generate paired-end libraries with 150 bp read length. The final results of the sequencing process were outputted as FASTQ files. A conventional short-read sequencing was performed by using the Qiagen Blood and tissue DNeasy to isolate genomic DNA from liver tissues according to the manufacturer’s description (Qiagen GmbH). The Paired-End Sequencing Sample Prep kit was used to prepare libraries from 5 µg of isolated gDNA according to the manufacturer’s description (Illumina). Either a 200 bp band or a 500 bp band was cut from the gel (libraries PE200 and PE500, respectively). After amplification the resulting libraries were analyzed with an Agilent Bioanalyzer 2100 DNA 1000 series II chip according to the manufacturer’s description (Agilent). Illumina Hiseq 2500 system will be used to perform short-read genome sequencing. Genomic libraries are paired-end sequenced with a read length of 150 nucleotides (150 bp). The results are genomic sequences in the form of FASTQ files. All-low quality reads (Q score < 30) were eliminated using Sickle^22^.

### De novo genome assembly

The de novo assembly of the 10x Genomics linked-read sequencing was performed using Supernova version 2.1.1^23^. FASTQ files were used as input when executing the software, and parameters were configured as recommended by the software guidelines. The assembly process was done using a computer workstation with 32 CPU cores and 512 GB RAM. The assembly of conventional short reads was carried out using SOAPdenovo2 (version 2.04) executing on the National Institutes of Health (NIH) Biowulf cluster (https://hpc.nih.gov/).

### Venom gland transcriptome data generation

Fresh tissue samples of the venom gland were harvested from a female *D. siamensis*. Illumina TruSeq RNA Sample Prep Kits were used for the RNA library preparation from these samples. The sequencing of the venom gland transcriptome was achieved using Illumina Hiseq 2500 system. Rcorrector^24^ and Cutadapt^25^ were used to correct sequencing errors and remove low-quality bases.

### Genome annotation

Annotations for the assembled *Daboia siamensis* scaffolds were achieved using the automated genome annotation pipeline MAKER^26^. This software performs ab initio gene prediction, protein and transcript alignment, repeat identification, and 5’ and 3’ UTR inference. The final gene models along with quality control statistics are provided by MAKER. Inputs for MAKER included the assembled *Daboia siamensis* genome, repeats identified by RepeatModeler (http://www.repeatmasker.org/RepeatModeler/) and Repclass^27^, proteins corresponding to Serpentes suborder (taxon identifier 8570) downloaded from UniProt database^28^, and transcriptome data of the venom gland. Ab initio genes and coding DNA sequences (CDSs) were predicted by MAKER.

### Scaffold quality assessment

The quality of scaffolds was assessed using BUSCO^29^ to estimate the genome completeness score. In this study, the Vertebrate near-universal single-copy orthologs (SCOs) database was selected as a search database for quality assessment.

### Venom protein identification using in-gel and in-solution digestion techniques

Venom proteins were separated by one-dimensional sodium dodecyl sulfate–polyacrylamide gel electrophoresis (1D SDS-PAGE). Briefly, 40 µg of crude venom was loaded onto bis–tris 12.5% acrylamide gels (Life Technologies). Gels were run at 200 V for 50 min, stained overnight with 0.1% Coomassie brilliant blue R-250, destained with water. Destained gels were scanned with the Odyssey infrared imaging system. Molecular weights were estimated based on protein standards. The gel was cut into 13 fractions from top to bottom. Each gel fraction was destained with 50% ACN/25 mM ammonium bicarbonate, reduced with 10 mM dithiothreitol (DTT) at 56 °C for 30 min, and alkylated with 55 mM iodoacetamide (IAA) at room temperature for 30 min in the dark. For trypsinization, 12.5 ng/µl trypsin solution was added to each sample and incubated for 60 min on ice. Excess trypsin was removed, an additional 50 µl of 25 mM ammonium bicarbonate was added to gel pieces, and samples were digested overnight at 37 °C. Following the overnight digestion, the samples were vortexed for 1 h at room temperature and sonicated in an ultrasonic bath for 3 min, and the tryptic peptides were extracted in 50 µl of 50% ACN/0.1% FA. The tryptic peptides were desalted with C18 StageTips. The samples were dried and stored at -80 °C for mass spectrometry analysis.

For in-solution digestion, 100 µg of crude venom was reconstituted in 8 M urea and added 10 mM DTT for reduction at 56 °C for 30 min. The sample was alkylated by adding 40 mM of IAA at room temperature for 30 min in the dark. Subsequently, 2 µg of trypsin was added and incubated overnight. On the following day, 10 µl of 100% FA was added, centrifuged at room temperature for 10 min, and then placed in a SpeedVac concentrator. The sample was reconstituted with 0.1% FA and fractionated by using Pierce high pH reversed-phase peptide fractionation kit (Thermo scientific). Peptides eluted from each fraction were dried and stored at − 80 °C for mass spectrometry analysis.

### Nano-liquid chromatography-tandem mass spectrometry and database searches

Peptides were separated by nano-liquid chromatography (EASY-nLC 1000, Thermo Fisher Scientific) coupled to a mass spectrometer (Q Exactive Plus Hybrid Quadrupole-Orbitrap, Thermo Fisher Scientific) through an EASY-Spray nanoelectrospray ion source (Thermo Fisher Scientific). The MS methods included a full MS scan at a resolution of 70,000 followed by 10 data dependent MS2 scans at a resolution of 17,500. The full MS scan range of 200–2000 m/z was selected, and precursor ions with the charge states of + 1 or greater than + 8 were excluded. Normalized collision energy of HCD fragmentation was set at 28%. Raw LC–MS/MS files were searched using PEAKS Studio version 8.5 against UniProt proteins corresponding to suborder Serpentes and the CDS database predicted by MAKER. A target-decoy approach was used to limit a false discovery rate (FDR) of the identified peptides to less than 1%. Parent and fragment monoisotopic mass errors were set at 10 ppm. Carbamidomethylation of cysteine (C) was used as a fixed modification. Oxidation (M), acetylation (protein N-term), phosphorylation (STY), and deamidation (NQ) were set as variable modifications. A maximum of 1 missed cleavage was allowed.

### De novo sequencing-based MS/MS spectra search

Additionally, raw LC–MS/MS files were searched by SMSNet^30^ using the venom gland transcriptome, UniProt proteins corresponding to suborder Serpentes concatenated with the CDS database as the reference database. This software is implemented based on deep learning models incorporated with de novo sequencing processes for the MS/MS spectra interpretation. The SMSNet-M model which recognizes variable oxidation of methionine was used. Precursor mass tolerance was set at 30 ppm. The false discovery rate was set at 5% at amino acid level. Low confidence amino acid positions were transformed into mass tags. Partially identified peptides and peptides containing isoleucine/leucine were searched against the reference databases. For example, a partially identified peptides HG(196.1203)TSACK will be matched to all reference sequences that start with HG, end with TSACK, and contain a combination of amino acids whose total weight is 196.1203 in the middle. Only those with unique hits were retained as the final identified peptides. For example, since 196.1203 is roughly equal to the combined mass of a valine and a proline, if both HGVPTSACK and HGPVTSACK are present in the reference database, the partially identified peptide HG(196.1203)TSACK would be discarded because we could not distinguish between the two possibilities. All CDSs with at least one mapped peptide were compared with search results provided by PEAKS Studio.

### Venomics studies using integrated genomics, transcriptomic, and proteomics data

*Daboia siamensis* toxin genes, transcripts, and proteins were identified by (a) annotation process by MAKER and (b) BLAST search of predicted proteins against UniProt toxins corresponding to suborder Serpentes. Hereby, both entries in Swiss-Prot (manually annotated) and TrEMBL (automatically annotated) were used. For each entry of BLAST search, only the top hit protein with e-value ≤ 1.00e−5 and %identities ≥ 70 was used for further analyses. Subsequently, identified toxins were clustered based on matching UniProt annotation into 2 major categories as described in the precedent research^16^: (1) toxins with enzymatic properties and (2) toxins with non-enzymatic properties. For the first group, there are 8 sub-categories: snake venom metalloproteinase (SVMP), snake venom serine protease (SVSP), phospholipase A_2_ (PLA_2_), phospholipase B (PLB), l-amino acid oxidase (LAAO), Kunitz-type serine protease inhibitor (KSPI), hyaluronidases, and nucleases (RNase, DNase, and phosphodiesterase). For the second group, there are 11 sub-categories according to the well-known non-enzymatic protein families in snake venoms: cysteine-rich secretory proteins (CRISPs), snaclecs, nerve growth factors (NGFs), natriuretic peptides, three-finger toxins (3FTs), sarafotoxins, cobra venom factors (CVFs), vascular endothelial growth factors (VEGFs), vespryns, waprins, and veficolins. Identified proteins were associated with their gene ontology (GO) terms using the “Retrieve/ID mapping” service provided by UniProt. By default, the GO term is not present in the mapping results. The option to show GO-related information must be selected first using the “edit column” button.

## Results and discussion

### Integrated multi-omics approach for comprehensive venomics profiling

**Figure 1:**
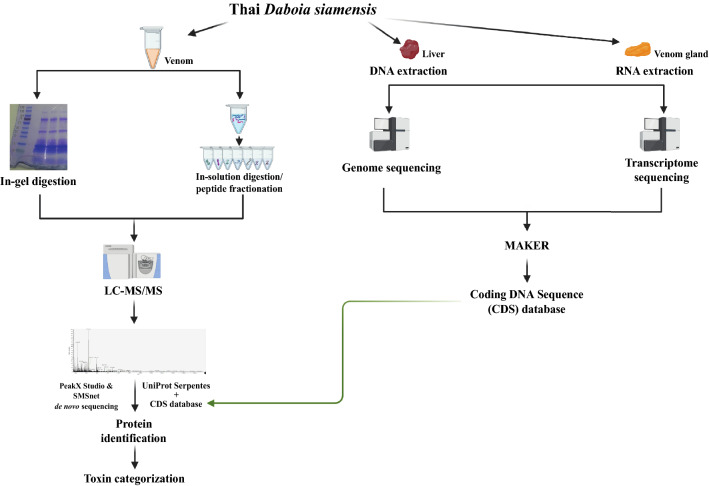
Workflow of our integrated multi-omics approach. The full-size figure of venom SDS-PAGE gel is shown in supplement Fig. S1.

In order to comprehensively profile the venome of *Daboia siamensis*, we used integrated multi-omics data that incorporate genomics, transcriptomics, and proteomics layers (Fig. 1). Genome and transcriptome data from *Daboia siamensis* liver and venom gland, respectively, were first integrated to generate a more correct and complete coding DNA sequence (CDS) database. Subsequently, this CDS database was used for proteomic search of the venom sample analyzed via mass spectrometry. In addition to the database search strategy, a de novo search strategy was also applied to increase the protein identification in the venom sample. Finally, toxin categorization was performed on the identified proteins. The details and results of each step in this multi-omics approach were described as follows.

### Genome sequencing

The sequencing process yielded 944.31 M paired-end reads. All raw reads were 150 bp in length with 69.38x coverage, exceeding the ideal coverage of 56x (Table 1). According to Supernova technical note^31^, higher coverage is occasionally advantageous in the assembly process. After trimming barcode sequences, the average length of reads was 138.5 bp, slightly lower than the ideal length at 140 bp. The estimated effective coverage of trimmed reads was 42.19x which is close to the ideal value. The percentage of Q30 bases in R2 reads and the median insert size were 76.81 and 361 bp, respectively. Both percentages were in the ideal ranges. Other measurements related to gDNA library are shown in the supplement Table S1.

**Table 1:** Statistics of raw sequenced reads used in the assembly process.

Measurement	Value from *Daboia siamensis* reads	Ideal value*
Number of reads	944.31 M	–
Read length	150 bp	–
Raw coverage	69.38x	~ 56x
Mean read length after trimming	138.5 bp	140 bp
Effective read coverage	42.19x	~ 42x
Fraction of Q30 bases in Read 2	76.81%	75–85%
Median insert size	361 bp	350–400 bp

*The ideal statistics are defined by Supernova^23^.

### De novo assembly

**Figure 2:**
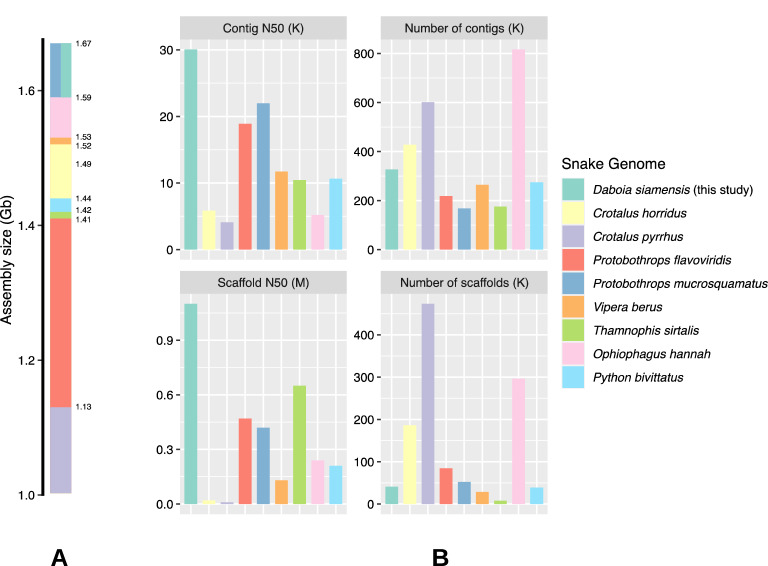
Comparisons of snake genome assemblies. The overall assembly sizes (**A**) and contig and scaffold statistics (**B**) of *Daboia siamensis* (this study), *Crotalus horridus*^38^, *Crotalus pyrrhus*^35^, *Protobothrops flavoviridis*^34^, *Protobothrops mucrosquamatus*^39^, *Vipera berus* (GenBank assembly accession: GCA_000800605.1), *Thamnophis sirtalis*^40^, *Ophiophagus Hannah*^33^, and *Python bivittatus*^19^.

The assembly process generated a 1.67 Gb *Daboia siamensis* genome with a scaffold N50 of 1.10 Mb. The total number of scaffolds was 40.97 K. Among these assembled scaffolds, 9.23 K were longer than 10 Kb. The overall GC content was 38.89% (Table 2). Other measurements related to assembled scaffolds are shown in supplement Table S2. Sequences of genome scaffolds were deposited at The National Center for Biotechnology Information (NCBI) repository (BioProject: PRJNA794313). For the assembly quality assessment, genome completeness scores of 88% and 8% were estimated by BUSCO^29^ based on complete and fragmented SCOs, respectively. The use of linked-read technology resulted in longer scaffolds compared to our study using only a conventional short-read technology. The assembly of short reads was carried out using SOAPdenovo^32^ which yielded 141 K scaffolds with N50 of 17 Kb, approximately 71 folds shorter than current scaffolds (Table S3). According to Fig. 2, the assembly sizes of published snake genomes range from 1.1 to 1.7 Gb. Contig and scaffold N50s of *Daboia siamensis* assembly are longer than other snake genomes such as *Python bivittatus*^19^, *Ophiophagus Hannah*^33^, *Protobothrops flavoviridis*^34^, and *Crotalus pyrrhus*^35^. However, when comparing *Daboia siamensis*’s scaffold N50 with the recently published genomes of *Naja naja*^36^ (scaffold N50 = 223.35 Mb) and *Pantherophis guttatus*^37^ (scaffold N50 = 16.79 Mb), our scaffold N50 is shorter. These recent studies employed and ensembled scaffolds from multiple state-of-the-art long-read technologies such as 10x linked-read, PacBio, Chicago, and Oxford Nanopore altogether, thus scaffolds with exceptionally long N50 can be achieved.

**Table 2:** Statistics of assembled scaffolds for genomic data.

Measurement	Value
Assembly size	1.67 Gb
Number of contigs	4.11 M
Number of scaffolds	40.97 K
Number of scaffolds with length ≥ 10 Kb	9.23 K
N50 contig size	30,074
N50 scaffold size	1.10 Mb
GC content of assembly	38.89%

### Annotation results

In total, 19,691 genes were predicted from *Daboia siamensis* assembled genome by using the MAKER annotation pipeline (Table 3). Regions of genes cover ~ 5% of the assembled genome. From these regions, 19,772 CDSs were identified which cover ~ 0.8% of the assembled genome and ~ 15.99% of gene regions. These CDSs were compiled into the “CDS database” for the mass spectrometry search as described in the “Venom proteome” section below. Note that the transcriptome data used in the annotation process was generated from the *Daboia siamensis* venom gland. Therefore, many annotated CDSs are related to snake venoms. The detailed annotation report from MAKER is in supplement Table S4.

**Table 3:** Number of genes, exons, introns, and mRNAs identified by MAKER.

Region of	Number of predicted regions	Total region length (Mb)
Genes	19,691	87.77
Exons	72,598	14.85
Introns	52,826	73.18
CDS	19,772	14.04

### Classification of venom CDS

**Figure 3:**
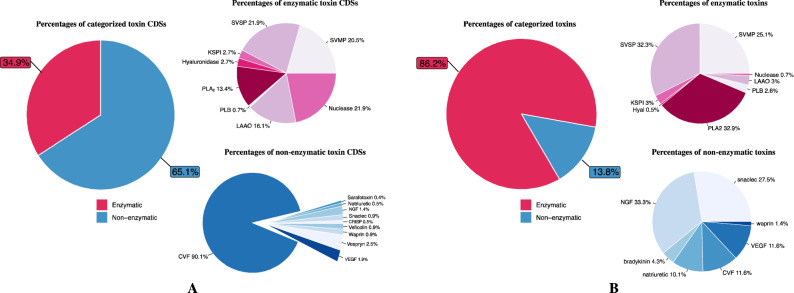
Percentages of CDSs (**A**) and proteins (**B**) categorized in enzymatic and non-enzymatic groups.

CDSs were categorized as described in “Methods”, “Venomics studies using integrated genomics, transcriptomic, and proteomics data” section (see supplement Table S5 for a full list of CDSs). From all 19,772 CDSs, 17,676 (~ 89.4%) can be annotated by using BLAST search and 915 CDSs can be categorized into two major toxin categories, enzymatic and non-enzymatic toxin groups (Fig. 3A). In the enzymatic group, there are 319 CDSs in total (34.9%). These CDSs were classified into 6 subgroups. The top-three common subgroups belong to SVSP, nuclease, and SVMP followed by LAAO and PLA_2_ subgroups. The least common subgroup is PLB which includes only 2 CDSs (2.8%). The non-enzymatic group includes 596 CDSs (65.1%). These CDSs were divided into 7 subgroups. The most common subgroup is CVF which includes 534 CDSs (90.1%). Only 18 CDSs were classified as vespryn (2.5%), and the remaining 8 subgroups include 44 CDSs (7.4%).

### Venom proteome

Proteins from *Daboia siamensis* venom gland were processed for mass spectrometry using two techniques: (1) SDS-PAGE followed by in-gel digestion and (2) in-solution digestion followed by high pH peptide fractionation to separate complex proteins and detect low abundance proteins. The generated MS/MS spectra were searched against the UniProt’s Serpentes reference database (taxon identifier 8570) concatenated with the CDS database. The search results yielded 10,131 and 4367 unique peptides from in-gel and in-solution digestion techniques, respectively. The combined list of peptides contains 17,163 unique peptides corresponding to 1662 unique proteins (supplement Table S6). Subsequently, all identified proteins were clustered into two categories: enzymatic and non-enzymatic proteins as described in “Methods”, “Venomics studies using integrated genomics, transcriptomic, and proteomics data” section. The proteome is dominated by the enzymatic group (86.2%), whereas only 13.8% of proteins belong to the non-enzymatic group (Fig. 3B). The enzymatic group consists of the following protein families: PLA_2_ 32.9%, SVSP 32.3%, SVMP 25.1%, LAAO 3%, KSPI 3%, PLB 2.6%, hyaluronidase 0.5%, and nuclease 0.7%. PLA_2_ family was found at the highest proportion among the enzymatic group, consistent with previous reports in Asian *Daboia siamensis* and Russell’s viper^41–43^. It is a family of enzymes that cleave glycerophospholipids to dissociate fatty acids tail. The pharmacological effect of this enzyme is well known, such as coagulant, hemorrhagic, hemolytic, edema forming activities, and neurotoxicity. Whereas neurotoxic effect is a common feature of *Daboia russelii* venom in Sri Lanka and some parts of India, it is less common in Asian *Daboia siamensis*. SVSP and SVMP are commonly found in this venom and have hemotoxic activity. To contrast the hemolytic activity, Op B et al. (2021) compared the venom of *Daboia siamensis* from Thailand, Myanmar, and Taiwan. They found that Myanmar *Daboia siamensis* venom has the most hemotoxic effect^44^. In the present study, we can detect LAAO toxin in the venom of *Daboia siamensis* from Thailand, similar to the reports from China and Myanmar^42,45^. In contrast, Changra TM (2020) reported that LAAO was not detected in venoms from Thailand and Taiwan *Daboia siamensis* venom^43^. LAAO does not directly cause hemorrhage, but it can cause an impairment of platelet aggregation via hydrogen peroxide production due to its catalyzing effects on the l-amino acid substrate. Interestingly, many proteins identified in this study have not been found in the published *Daboia siamensis* protein databases and reports, as shown in Table 4. For example, this study first discovered the enzyme hyaluronidase-1 (CDS ID 16116 with 62% sequence coverage) as a component of *Daboia siamensis* venom. The pairwise alignment showed that *Daboia siamensis* hyaluronidase-1 is 95.4% identical to Cerastes hyaluronidase-1 sequence (UniProt accession: A3QVN3, see Fig. S2). Hyaluronidase-1 enzyme degrades hyaluronic acid, a glycosaminoglycan commonly found in the extracellular matrix, to promote the local hemorrhagic effect. It is called a “spreading factor” because it promotes the spread of the toxin through the tissues and blood circulation of the prey. This enzyme could play an important role in destroying tissue at the bite site in the victim^46^. PLB, another rare toxin, was identified with 43% sequence coverage against CDS ID 14593. PLB is a protein in the phospholipase superfamilies. PLB toxin in *Pseudechis colletti* venom was reported to have hemolytic and cytotoxic activities in vitro in human erythrocytes^47^.

**Table 4:** Potential venom proteins identified from a different source of *Daboia siamensis* specimens.

Venom category	Protein name	CDS ID	Source of specimen						Pathophysiological effects
			Thailand (this study)	Thailand^43^	Indonesia^43^	Taiwan^42^	China^42^	Myanmar^41^	
KSPI	Kunitz-type serine protease inhibitor C6	6337	✓	✓	✓	✓	✓	✓	Coagulopathy
PLA_2_	Acid PLA_2_	9491	✓	✓	✓	✓	✓	✓	Coagulopathy, neurotoxicity, edema
	Basic PLA_2_	11762	✓	✓	✓	✓	✓	✓	
	Phospholipase A2 inhibitor	2357	✓						
	Phospholipase A2 inhibitor subunit gamma B	3191	✓						
PLB	Phospholipase B	14593	✓						Hemotoxic activity
SVSP	Vaa serine proteinase homolog 1	8750	✓						Hemotoxic activity, coagulopathy
	Complement factor I	6095	✓						
	Peptidase S1 domain-containing protein	4112	✓						
	Factor V activator RVV-V gamma	18736	✓	✓	✓	✓		✓	
	Serine protease VLSP-1	370	✓					✓	
	Serine protease VLSP-3	18982	✓	✓	✓			✓	
	Beta-fibrinogenase-like	2680	✓	✓	✓			✓	
	Alpha-fibrinogenase-like	10579	✓	✓	✓	✓		✓	
SVMP	Coagulation factor X-activating enzyme heavy chain protein	2879	✓	✓	✓	✓		✓	Hemotoxic activity
	Snaclec coagulation factor X-activating enzyme light chain 1	4878	✓	✓	✓	✓		✓	
	Antihemorrhagic factor cHLP-B protein	18915	✓						
	Antihemorrhagic factor jMSF protein	3892	✓						
LAAO	l-amino-acid oxidase	14325	✓	✓	✓	–	✓	✓	Coagulopathy
	Amine oxidase	3374	✓						
Nuclease	Deoxyribonuclease II	8825	✓						Hemotoxic activity
	Angiogenin-2	437	✓						
Snaclec	C-type lectin-like protein subunit 2	4878	✓	✓	✓	✓	✓	✓	Hemorrhage
	C-type lectin snaclec-1	15452	✓						
CVF	Actin-depolymerizing factor	13591	✓						Bleeding, complications
NGF	Beta-nerve growth factor	4415	✓						
VEGF	Snake venom vascular endothelial growth factor toxin VR-1	9987	✓	✓	✓	✓	✓	✓	
Amino peptidase	Xaa-Pro aminopeptidase 2	6377	✓			✓	✓	✓	Hypertension
	Aminopeptidase	10654	✓					✓	
	Tripeptidyl aminopeptidase	6651	✓						
Cathepsin	Cathepsin B	1357	✓						Procoagulant
	Cathepsin H	6109	✓						
Waprin	WAP, follistatin/kazal, immunoglobulin, kunitz and netrin domain containing 2	16646	✓						Anti-microbacterial
Hyaluronidase	Hyaluronidase-1	16116	✓						Hemorrhage, inflammation

**Protein digestion methods for mass spectrometry**									
In-solution digestion			✓	✓	✓				
In-gel digestion			✓			✓	✓		
In-gel digestion (2D gel electrophoresis)								✓	

**Figure 4:**
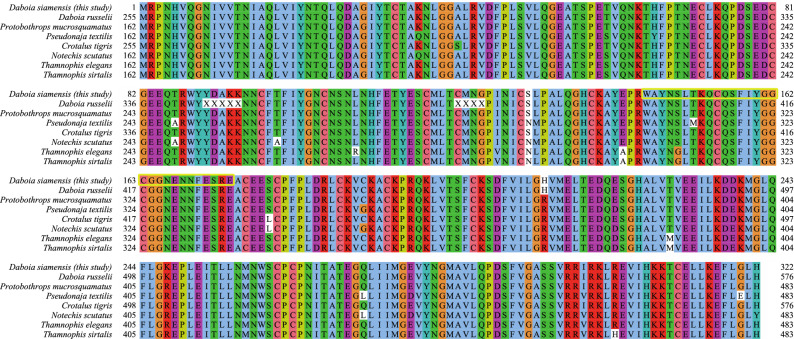
Multiple alignment of waprin protein from various venomous snakes. The NCBI reference sequence/GenBank accession numbers for *Daboia russelii*, *Protobothrops mucrosquamatus*, *Pseudonaja textilis*, *Crotalus tigris*, *Notechis scutatus*, *Thamnophis elegans*, and *Thamnophis sirtalis* waprins are ASU45069, XP_029139923, XP_026557480, XP_039199862, XP_026523009, XP_032094882, and XP_013912228, respectively. The single peptide of *Daboia siamensis* waprin identified in this study is highlighted in the yellow box.

The non-enzymatic group consists of NGF 33.3%, snaclec 27.5%, VEGF 11.6%, CVF 11.6%, natriuretic peptides 10.1%, bradykinin 4.3%, and waprin 1.4% (Fig. 3B). Snaclec is found in Asian *Daboia siamensis* venom. It has been reported to possess platelet-modulating activity that enhances the activity of SVMP to induce hemorrhage^48^. Whereas VEGF, CVF, and NGF show potential in inducing hypotension and enhancing vascular permeability leading to bleeding complications^49^. We also found waprin toxin, a rare toxin that has not been reported in *Daboia siamensis*, but reported in another Russell's viper species, *Daboia russelii* (GenBank accession: ASU45069). We identified a single peptide with high confidence (with various post-translational modifications, see Table S6), and the CDS of this protein is highly conserved across venomous snake species (Fig. 4). This protein exhibits proteinase inhibitor activity with potent antimicrobial activity^50^. We summarize the pathophysiological effects of all toxins identified in this study in Table 4.

**Figure 5:**
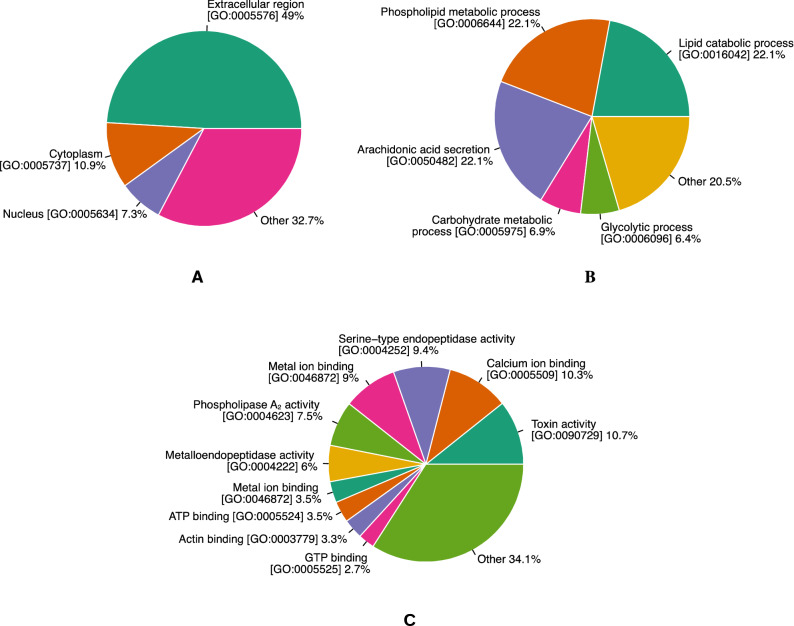
Percentages of functional group-based GO terms calculated from *Daboia siamensis* venom proteome according to cellular component terms (**A**), biological process terms (**B**), and molecular function terms (**C**).

All proteins identified in the venom were assigned to functional group-based Gene Ontology (GO) terms using the “Retrieve/ID mapping” service provided by UniProt database as described in “Methods”, “Venomics studies using integrated genomics, transcriptomic, and proteomics data” section. Figure 5 shows GO annotation of putative *Daboia siamensis* venom proteome. Most of the identified venom proteins (49%) seem to function in extracellular regions since SVMP, SVSP, LAAO, PLA_2_, and KSPI are located in extracellular regions (Fig. 5A). The top-five GO molecular function terms were annotated as toxin activity, calcium ion binding, serine-type endopeptidase activity, metal ion binding, and phospholipase A_2_ activity. These terms are consistent with the activities of component proteins shown in the venom proteome (Fig. 3B).

### Venom proteins identified by de novo sequencing-based MS/MS spectra searching

A total of 903 CDSs were identified by SMSNet (Table S7). Compared to the search results generated by PEAKS Studio, 175 proteins are overlapped (Table S8). Interestingly, 728 CDSs were solely identifiable by SMSNet. One of these CDSs is identified as *Daboia siamensis* apoptosis-inducing protease (DSAIP), a P-III class SVMP, first discovered in Burmese *Daboia siamensis*^41^ (UniProt accession A0A2H4Z2X4) and also found in China^42^ and Taiwan^51^. DSAIP protein is a homolog of *Vipera lebetina* apoptosis-inducing protease (VLAIP) which is also found in Russell's vipers endemic to Sri Lanka^52^ and Pakistan^53^ but not found in specimens from Western Indian^54^ and Southern Indian^55^.

**Figure 6:**
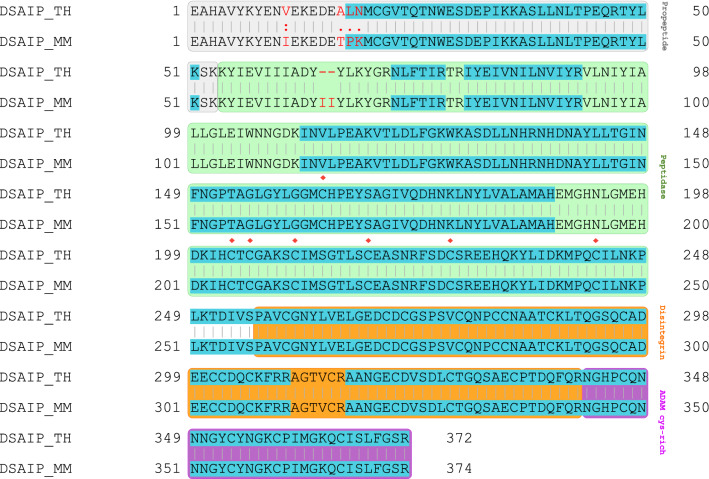
Pairwise sequence alignment of Thai (TH) and Myanmar (MM) DSAIP proteins. The grey, green, orange, and purple boxes denote regions of propeptide, peptidase, disintegrin, and ADAM cys-rich, respectively. Alphabets in red indicate differences between Thai and Myanmar DSAIP proteins. Blue-highlighted alphabets are regions covered by SMSNet search results. Red diamonds denote conserved cysteine residues.

Peptide sequences identified from SMSNet also validate the difference between Thai and Burmese DSAIP proteins as illustrated in the pairwise sequence alignment (Fig. 6). Amino acids leucine and asparagine at positions 17 and 18 of Thai DSAIP are consistent with proteomics results (Table S7). This may indicate one of the divergences between Thai and Myanmar DSAIP proteins. All differences at positions 11, 17, 18, and 19 are located in the propeptide region. However, sequences in peptidase, disintegrin, and ADAM cysteine-rich regions are identical. The majority of P-III class SVMPs consists of six conserved cysteinyl residues in the peptidase region, but for DSAIP there are seven conserved cysteinyl residues. The seventh cysteinyl residue is involved in the apoptosis-inducing activity^56^. In this study, seven conserved cysteinyl residues were identified in the peptidase region (Fig. 6).

In addition, many proteins identified by SMSNet have not been reported as a component of *Daboia siamensis* venom (Table S9). Similar to the use of PEAK Studio, a number of proteins were detectable when using CDSs as a reference database but undetectable when using the conventionally UniProt’s Serpentes reference database. These findings support the use of integrated multi-omics data as an improved approach for discoveries in venomics studies.

## Conclusion

This study incorporates genomics, transcriptomics, and proteomics data to study venomics of *Daboia siamensis.* Using integrated multi-omics data, we found numerous proteins that have not been reported as *Daboia siamensis* venom components such as hyaluronidase-1, phospholipase B, and waprin. Our findings indicate that the use of integrated multi-omics data is advantageous for venomics studies. The integrated multi-omics data along with our discoveries will be valuable not only for antivenom production but also for the development of novel therapeutics.

## Supplementary Information

Supplementary Figure S1.

Supplementary Figure S2.

Supplementary Table S1.

Supplementary Table S2.

Supplementary Table S3.

Supplementary Table S4.

Supplementary Table S5.

Supplementary Table S6.

Supplementary Table S7.

Supplementary Table S8.

Supplementary Table S9.

## Data Availability

The mass spectrometry proteomics data have been deposited to the ProteomeXchange Consortium via the PRIDE partner repository with the dataset identifier PXD030613. Genome data have been deposited at The National Center for Biotechnology Information (NCBI) repository (BioProject: PRJNA794313).
