# Supplementary figures and images for: Identification of Daboia siamensis venome using integrated multi-omics data
Source: Sci Rep. 2022 Jul 30;12:13140. doi: 10.1038/s41598-022-17300-1 (PMC9338987; doi:10.1038/s41598-022-17300-1)

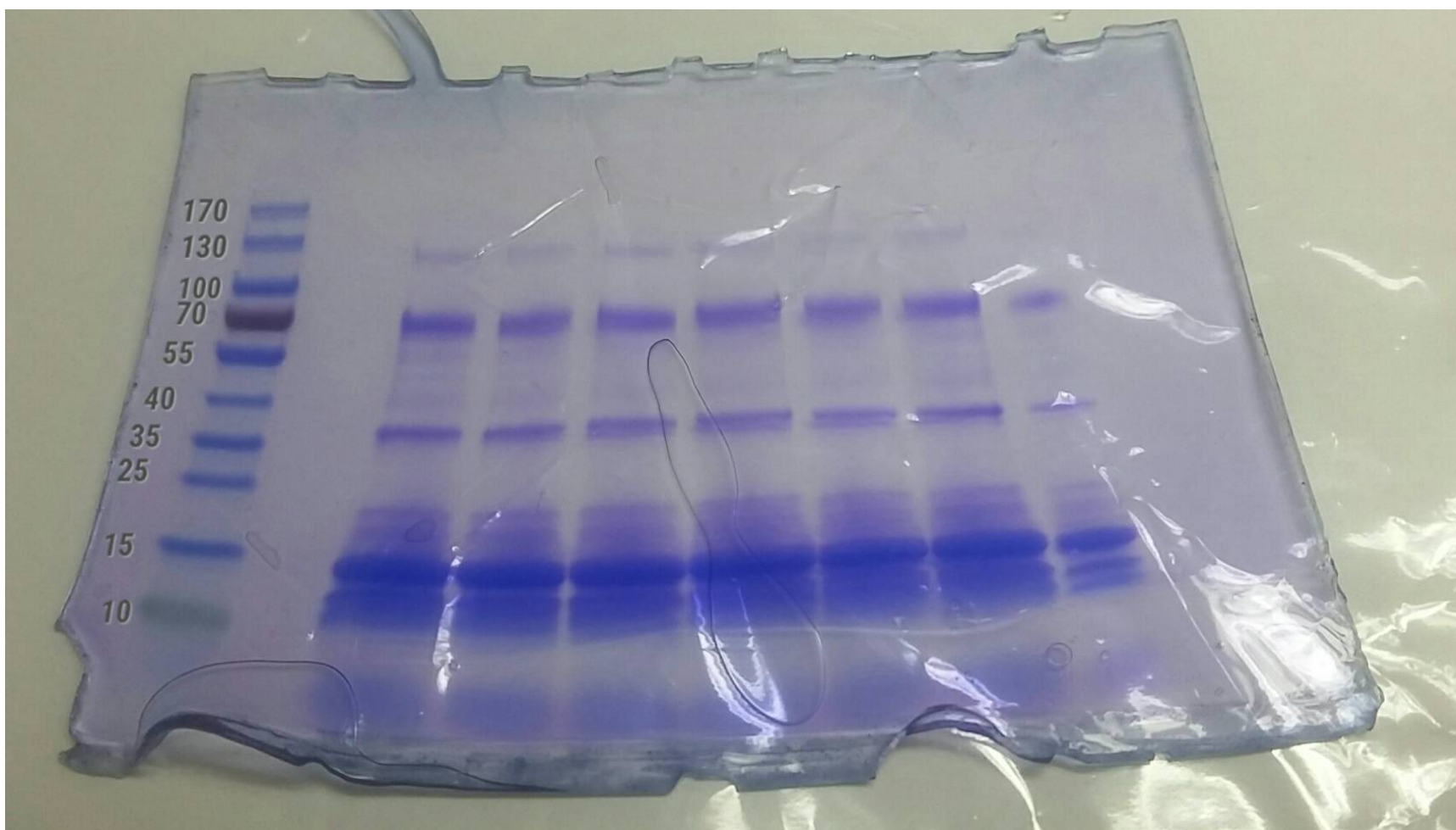

**Figure S1.** Venom SDS-PAGE gel. Venom sample was loaded for 7 lanes.

Supplement: Supplementary file 1 — Supplementary Figure S1. [file 41598_2022_17300_MOESM1_ESM.pdf]
