# Supplementary material for: Identification of Daboia siamensis venome using integrated multi-omics data
Source: Sci Rep. 2022 Jul 30;12:13140. doi: 10.1038/s41598-022-17300-1 (PMC9338987; doi:10.1038/s41598-022-17300-1)
Supplement: Supplementary file 2 — Supplementary Figure S2. [file 41598_2022_17300_MOESM2_ESM.pdf]

```
CDS 16116    1      MQAKAPMYPNEPFLVFWNAPTTQCRMRYKVDLDLKTFHIVTNANDSLSGSAVTIFYPNHL     60
                    MQAKAPMY NEPFLVFWNAPTTQCR+RYKVDLDLKTFHIV+NANDSLSGSAVTIFYPNHL
A3QVN3       24     MQAKAPMYRNEPFLVFWNAPTTQCRLRYKVDLDLKTFHIVSNANDSLSGSAVTIFYPNHL     83

CDS 16116    61     GVYPHIDDRGHFFHGIIPQNESLTKHLDKSKSDINRIIPLNTFHGLGVIDWENWRPQWDR     120
                    GVYPHIDDRGHFFHGIIPQNESLTKHL+KSKSDINRIIPL  FHGLGVIDWENWRPQWDR
A3QVN3       84     GVYPHIDDRGHFFHGIIPQNESLTKHLNKSKSDINRIIPLKAFHGLGVIDWENWRPQWDR     143

CDS 16116    121    NWGSKNVYRNRSIQFAKVLHPQLSEDKIRRLAKKQYEKAAKSFMRDTLLLAEEMRPNGYW     180
                    NWGSKNVYRNRSIQFA+ LHP+LSEDKIRRLAKK+YEKAAKSFMRDTLLLAEEMRP+GYW
A3QVN3       144    NWGSKNVYRNRSIQFARDLHPELSEDKIRRLAKKEYEKAAKSFMRDTLLLAEEMRPDGYW     203

CDS 16116    181    GYYLYPDCQNYDYKTKGDQYTGKCPEIEMSRNDQLLWLWRDSTALFPNVYLEIILRSSEN     240
                    GYYLYPDCQNYDYKTKGDQYTGKCPEIEMSRNDQLLWLWRDSTALFPNVYLEIILRSS+N
A3QVN3       204    GYYLYPDCQNYDYKTKGDQYTGKCPEIEMSRNDQLLWLWRDSTALFPNVYLEIILRSSDN     263

CDS 16116    241    ALKFVHHRLKESMRIASMAREDYALPVFAYARPFYAYTFEPLTQ                     284
                    ALKFVHHRLKE+MRIASMAREDYALPVFAYARPFYAYTFEPLTQ
A3QVN3       264    ALKFVHHRLKEAMRIASMAREDYALPVFAYARPFYAYTFEPLTQ                     307

E-value: 0.0
Score: 1494
Ident.: 95.4%
Positives : 98.6%
Query Length: 290
Match Length: 449
```

Figure S2. Pairwise alignment of hyaluronidase-1 proteins.
