# Supplementary material for: Identification of Daboia siamensis venome using integrated multi-omics data
Source: Sci Rep. 2022 Jul 30;12:13140. doi: 10.1038/s41598-022-17300-1 (PMC9338987; doi:10.1038/s41598-022-17300-1)
Supplement: Supplementary file 3 — Supplementary Table S1. [file 41598_2022_17300_MOESM3_ESM.pdf]

Table S1. Statistics of raw genomic reads used in the assembly process

Measurement	Value	Ideal Value†
Number of reads	944.31 M	-
Read length	150 bp	-
Mean read length after trimming	138.5 b	140 b
Raw coverage	69.38 x	~56 x
Effective read coverage	42.19 x	~42x for raw 56x
Fraction of Q30 bases in read 2	76.81 %	75-85%
Median insert size	361 b	350-400 b
Fraction of proper read pairs	87.93%	>=75%
Fraction of barcodes used	1	-
Estimated genome size	2.04 Gb	-
Genome repetitivity index	15.49%	-
High AT index	0.07%	-
Dinucleotide content	0.44%	-
Molecule count extending 10 kb on both sides	17.06	10 kb
Mean distance between heterozygous SNPs	1.72 Kb	-
Fraction of reads that are not barcoded	6.31%	-
N50 reads per barcode	744	-
Fraction of reads that are duplicates	28.48%	-
Nonduplicate and phased reads	37.83%	45-50%

†the ideal statistics are define by Supernova [1].

References

1 Weisenfeld, N. I., Kumar, V., Shah, P., Church, D. M. & Jaffe, D. B. Direct determination of diploid genome sequences. *Genome Res* **27**, 757-767, doi:10.1101/gr.214874.116 (2017).
