# Supplementary material for: Identification of Daboia siamensis venome using integrated multi-omics data
Source: Sci Rep. 2022 Jul 30;12:13140. doi: 10.1038/s41598-022-17300-1 (PMC9338987; doi:10.1038/s41598-022-17300-1)
Supplement: Supplementary file 4 — Supplementary Table S2. [file 41598_2022_17300_MOESM4_ESM.pdf]

Table S2. Contig and scaffold statistics of this study

Measurement	Value	Description
Number of scaffolds	40.97 K	number of scaffolds
Long scaffolds	9.23 K	number of scaffolds (length >= 10 kb)
Contig N50	30.07 Kb	N50 contig size
Scaffolds N50	1.10 Mb	N50 scaffold size
Gap 10KB	3.71%	% of long gap (gap length >= 10 kb)
Assembly size	1.67 Gb	assembly size
Assembly size of long scaffolds	1.55 Gb	assembly size of long scaffolds (length >= 10 kb)
Assembly GC content	38.89%	GC content of assembly
