# Supplementary material for: Identification of Daboia siamensis venome using integrated multi-omics data
Source: Sci Rep. 2022 Jul 30;12:13140. doi: 10.1038/s41598-022-17300-1 (PMC9338987; doi:10.1038/s41598-022-17300-1)
Supplement: Supplementary file 5 — Supplementary Table S3. [file 41598_2022_17300_MOESM5_ESM.pdf]

Table S3. Contig and scaffold statistics of short and linked- read technology

Sequencing Technology	Assembler Software	Scaffold Statistics	
		N50	#Scaffold
10x linked-read	Supernova	1.10 Mb	40.97 K
Illumina HiseqX	SOAPdenovo2	16.76 Kb	141.16 K
