# Supplementary material for: Identification of Daboia siamensis venome using integrated multi-omics data
Source: Sci Rep. 2022 Jul 30;12:13140. doi: 10.1038/s41598-022-17300-1 (PMC9338987; doi:10.1038/s41598-022-17300-1)
Supplement: Supplementary file 6 — Supplementary Table S4. [file 41598_2022_17300_MOESM6_ESM.pdf]

Table S4. Result of annotation process reported from MAKER [1].

Measurement	Value
Number of genes	19691
Number of mRNAs	19772
Number of exons	72598
Number of introns	52826
Number of CDS	19772
Overlapping genes	33
Contained genes	10
Total gene length	87768215
Total mRNA length	87927865
Total exon length	14851466
Total intron length	73182051
Total CDS length	14038365
Shortest gene	3
Shortest mRNA	3
Shortest exon	1
Shortest intron	20
Shortest CDS	3
Longest gene	67598
Longest mRNA	67598
Longest exon	11561
Longest intron	17203
Longest CDS	13350
mean gene length	4457
mean mRNA length	4447
mean exon length	205
mean intron length	1385
mean CDS length	710

References

1 Holt, C. & Yandell, M. MAKER2: an annotation pipeline and genome-database management tool for second-generation genome projects. *BMC Bioinformatics* 12, 491, doi:10.1186/1471-2105-12-491 (2011).
